# Bibliometric analysis of recent sodium channel research

**DOI:** 10.1080/19336950.2018.1511513

**Published:** 2018-09-29

**Authors:** Dongyi Zhao, Jianing Li, Corey Seehus, Xuan Huang, Meimi Zhao, Shiqi Zhang, Wuyang Wang, Hong-Long Ji, Feng Guo

**Affiliations:** a Department of Pharmaceutical Toxicology, School of Pharmacy, China Medical University, Shenyang, China; b FM Kirby Institution, Neurobiology Department, Harvard University, Boston, MA, USA; c Department of Cellular and Molecular Biology, University of Texas Health Science Center at Tyler, Tyler, TX, USA; d Jiangsu Province Key Laboratory of Anesthesiology, Xuzhou Medical University, Xuzhou, China

**Keywords:** Bibliometric, CiteSpace V, sodium channel, WoSCC

## Abstract

Although sodium channels have been a hot multidisciplinary focus for decades and most of nerve system drugs worked on alerting sodium channel function, the trends and future directions of sodium channel studies have not been comprehensive analyzed bibliometrically. Herein, we collected the scientific publications of sodium channels research and constructed a model to evaluate the current trend systematically.

Publications were selected from the Web of Science Core Collection (WoSCC) database from 2013 to 2017. Microsoft Excel 2016, Prism 6, and CiteSpace V software were used to analyze publication outputs, journal sources, countries, territories, institutions, authors, and research areas. A total of 4,275 publications on sodium channel research were identified. PLoS ONE ranked top for publishing 170 papers. The United States of America had the largest number of publications (1,595), citation frequency (19,490), and H-index (53). S. G. Waxman (62 publications) and W. A. Catterall (585 citations) were the most productive authors and had the greatest co-citation counts. This is the first report that shows the trends and future development in sodium channel publications, and our study provides a clear profile for the contribution to this field by countries, authors, keywords, and institutions.

## Introduction

Sodium channels are integral membrane proteins that form ion channels, conducting sodium ions (Na^+^) through a cell’s plasma membrane [1]. They are classified according to the trigger that opens the channel for such ions, which can either comprise “Voltage-gated sodium channels” (“VGSCs” or “Na_V_ channels”) or the binding of a substance (a ligand) to the channel (ligand-gated sodium channels) [2].

Sodium channels were first discovered in the squid giant axon using the voltage clamp procedure [3]. The voltage-dependent activation of sodium channels initiates electrical signals, which control nerve actions [4]. In excitable cells such as neurons, myocytes, and certain types of glia, sodium channels are responsible for the rising phase of action potentials. These channels go through three different states: resting, active, and inactive. Although neither resting nor inactive states allow the ions to flow through the channels, a difference exists with respect to their structural conformation [5].

Furthermore, specific sodium channel perturbations affect many neurological diseases including epilepsy, Alzheimer’s disease, stroke, migraine and other headache diseases, multiple sclerosis, Parkinson’s disease, neurological infections, brain tumors, etc [6].

Research on sodium channels began with the discovery of the sodium channel protein and the claim that sodium channels were composed of primary pore-forming subunits with auxiliary subunits [7]. Thereafter, considerable research was conducted into fast and slow inactivation through its use on drug receptor sites in sodium channels [8]. There has also been marked improvement in research into the crystal structure of sodium channels [9], with the identification of the voltage-gated ion channel protein [10].

A bibliometric study is an effective way to calculate the longitudinal trends in research activity and clarify the connection between relevant research institutions[11]. Bibliometrics is considered an application of statistics and is often used in mathematics; it comprises analyzes of written publications, such as books and journal articles, in a given field to ascertain various properties[12]. In bibliometric research, the literature system and literature metrology characteristics are used as the research objects and the literature is analyzed both quantitatively and qualitatively[13].

However, to date, no bibliometric studies regarding the trends in sodium channel research activity over the past few decades have been published. Although bibliometrics can measure publication trends in many disciplines, no related analysis of the research progression for sodium channels has been published and there has been a consequent lack of attention to this field.

The purpose of our study was to provide researchers with some direction regarding sodium channel research using bibliometrics. To achieve this, we used bibliometrics to dissect the characteristics of scientific articles on the Web of Science (Thomson Reuters Company) into several components so that subjective factors are minimalized and then analyzed the overall publication trends. Our study confirmed that the United States has contributed greatly to sodium channel research, while S.D. Dib-Hajj, K. Dong, and Y. Yang may be good candidates for future research collaboration. We also identified “Protein,” “pyrethroid resistance,” “function mutation,” “sodium channel gene,” and “arrhythmia” as the latest research frontiers, and related studies may become pioneering in this field in the coming years.

## Materials and methods

### Data resource and search strategy

The data for analysis were extracted from the Science Citation Index Expanded (SCI-EXPANDED) of Thomas Reuters Web of Science Core Collection (WoSCC) bibliographic database. To ascertain the trend in publications, we collected five years’ worth of data from 2013 to 2017. The data were downloaded directly from the database as secondary data without further animal experiments; therefore, no ethical approval was required.

The data search was conducted on 31 December 2017 and collected in one day to avoid any potential bias due to the daily updating of the database. The search keywords entered into the database were as follows: TS = (“sodium channel”) OR TS = (“sodium channels”) OR TS = (“sodium ion channel”)) AND LANGUAGE: (English) AND DOCUMENT TYPES: (ARTICLE OR PROCEEDINGS PAPER).

### Data collection

The txt data were downloaded from Web of Science and imported to Microsoft 2016, GraphPad Prism 6 (GraphPad Software Inc., San Diego, CA), CiteSpace V. All data were analyzed both quantitatively and qualitatively.

### Statistical analysis

Bibliometrics is an essential tool to measure the output of an author, institution, journal, or country; it measures relevant parameters including impact factor, quantity, and the total number of citations of published articles [14].

The retrieved documents were sorted by the number of citations they had received over a period of five years from highest to lowest [14]. The “H-index” has been indicated as a trustworthy method of predicting future research, and comprises the time-cited publications belonging to a given country compared to the number of times those publications are at least co-cited.

To stratify different countries’ publications by world region, we used Microsoft Excel 2016 (Redmond, Washington, United States) to create a pie-chart. A comparison of publication quantity, citations, and H-indices between different countries were organized using GraphPad Prime 6. The statistical results were then displayed using CiteSpace V (Drexel University, Philadelphia, the United States), a visualization software for analyzing data using network modeling [15]. Finally, the consequence and number of co-cited authors and co-cited references were calculated using VOSviewer (Leiden University, Leiden, Netherlands).

## Results

### General information and annual publication output

**Figure 1.**
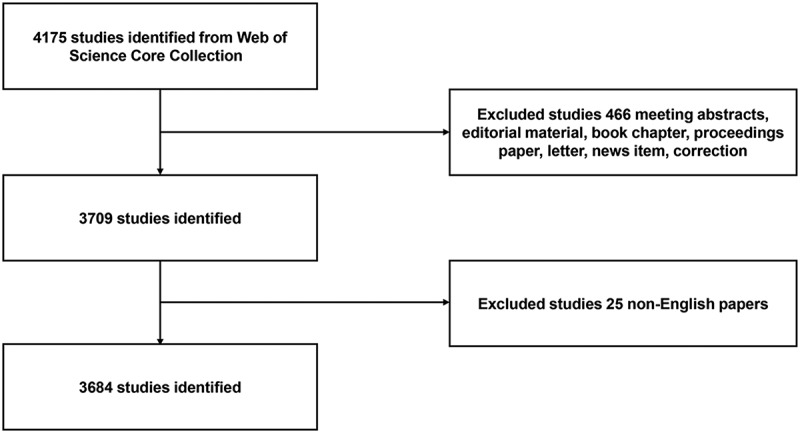
Flow diagram of sodium channel researches inclusion.

**Figure 2.**
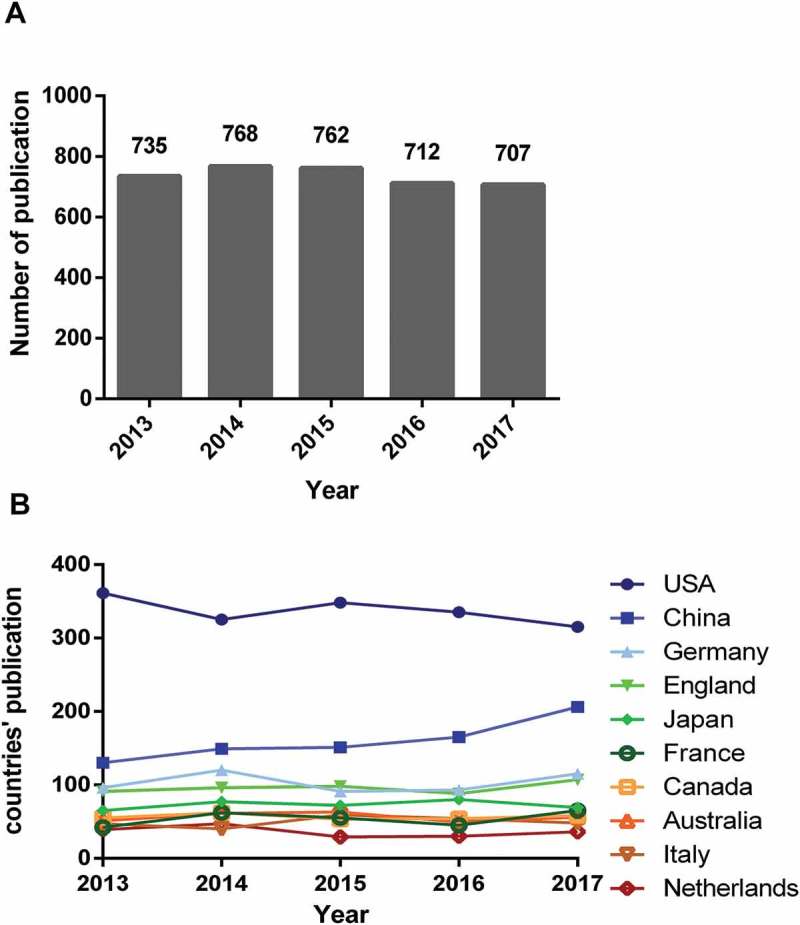
Publication outputs and growth prediction. (a). The number of annual publications on sodium channel research from 2013 to 2017; (b). The line chart of different countries on sodium channel publications trend.

A total of 4175 publications were retrieved from the Web of Science Core Collection, most of which comprised research articles and reviews (Table 1). Of the articles that were retrieved, 98.83% were written in English (see Table 2 for the full spectrum of languages used). The search criteria (sodium channel, sodium channels, sodium ion channel) produced 3684 pieces of literature and the flowchart of literature including these terms is shown in Figure 1. The number of publications remained steady in general but decreased slightly after 2014. Moreover, the overall trend increased from 735 papers in 2013 to 768 papers in 2014 but reduced from 762 papers in 2015 to 707 papers in 2017 (Figure 2(a)).

**Table 1.** The type of the retrieved document.

Rank	Type of document	Frequency N = 4175	Percent
1	Article	3056	73.20
2	Review	605	14.49
3	Meeting Abstract	328	7.86
4	Editorial Material	78	1.87
5	Book Chapter	45	1.08
6	Proceedings Paper	29	0.69
7	Correction	13	0.31
8	Letter	12	0.29
9	News Item	5	0.12
10	Biographical Item	4	0.10

**Table 2.** The type of the languages encountered in retrieved documents.

Rank	Language	Frequency N = 4175	Percent
1	English	4126	98.83
2	German	15	0.36
3	French	9	0.22
4	Spanish	9	0.22
5	Russian	7	0.17
6	Hungarian	3	0.07
7	Chinese	2	0.05
8	Japanese	2	0.05
9	Czech	1	0.02
10	Polish	1	0.02

While the trend of world sodium channel research publications remained stable in these years, several countries had variable publication data outputs. For example, the USA’s publication output decreased between 2015 and 2017. In contrast, China’s research publications increased continuously from 2013. Apart from the Netherlands, other countries also continued to increase their output compared to 2013 (Figure 2(b)).

### Citation and H-index analysis

**Figure 3.**
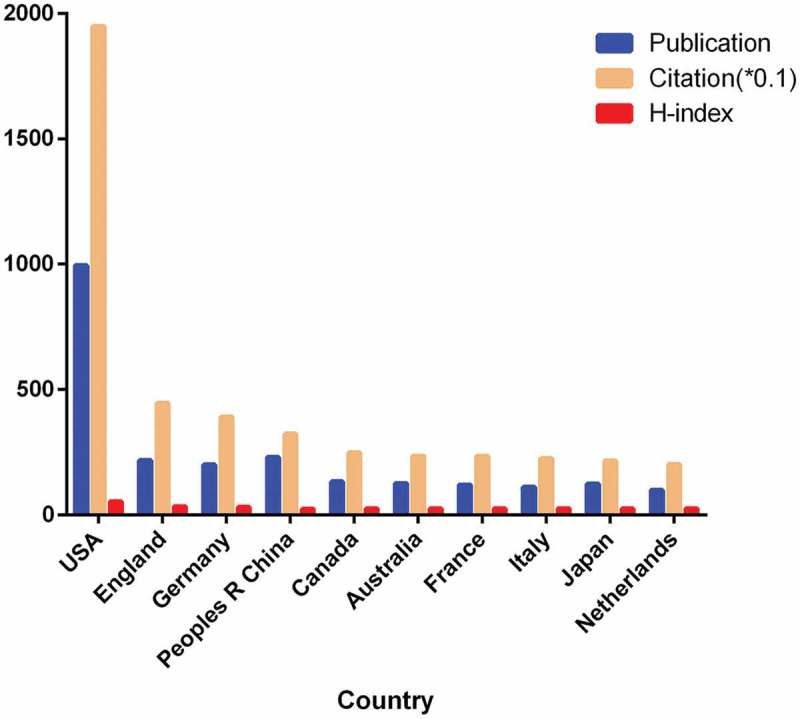
The publications, citation counts (× 0. 1), and H-index in the top 10 countries.

Based on our analysis of the Web of Science database, all papers related to sodium channels were cited 51,471 times from 2013 (38,707 times excluding self-citations). The average citation frequency of each paper was 9.81 times. Papers from the United States received the highest number of citations (19,490), accounting for 37.87% of the total number. The H-index of papers from the USA was 53. England ranked second with 4464 citations (8.67%) and a H-index of 33. Germany then followed with a citation frequency of 3909 (7.59%) and a H-index of 31 (Figure 3).

### Active countries and institutions

**Figure 4.**
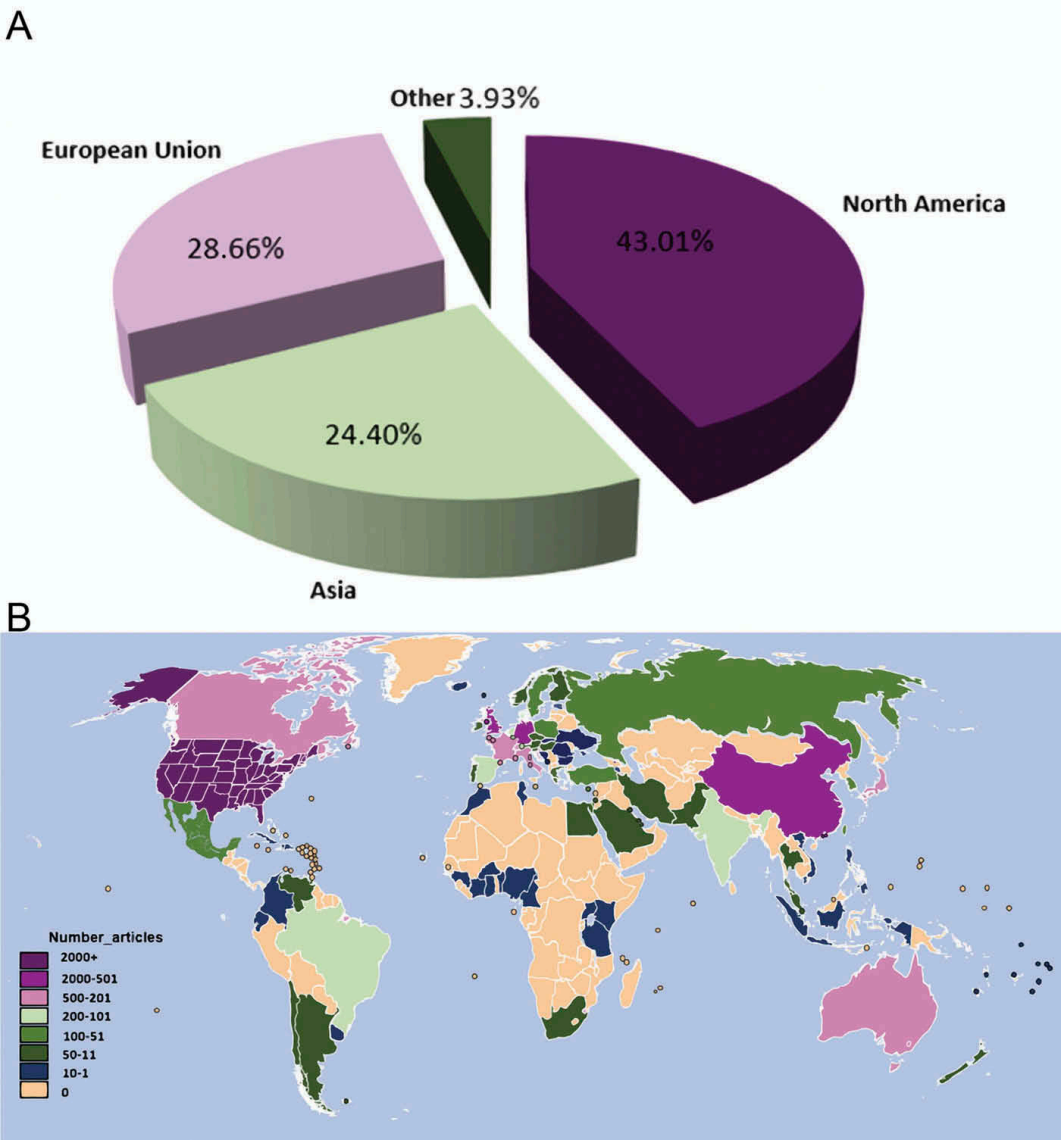
Worldwide publications analysis (a). The pie chart of research on sodium channel by different regions; (b). Geographic distribution of different countries.

**Figure 5.**
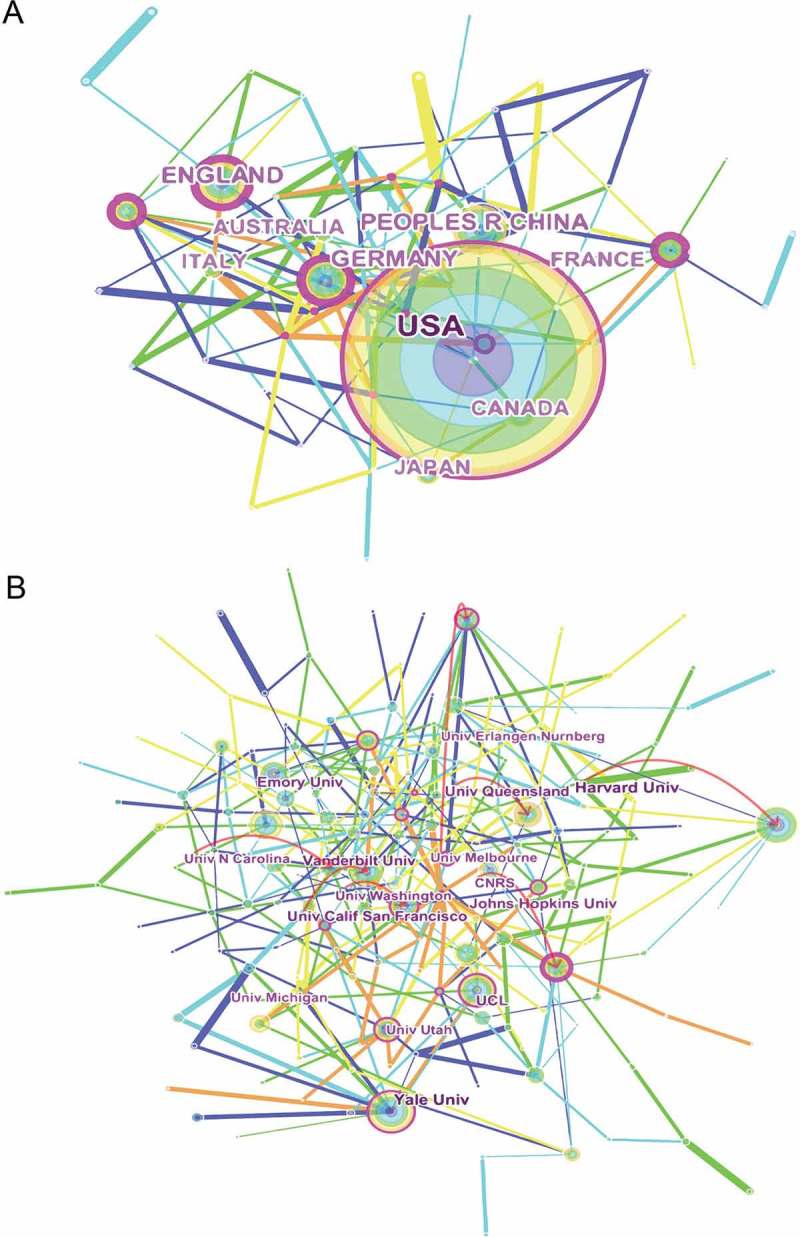
The analysis of countries and institutions. (a). Network of countries/territories engaged in sodium channel research; (b). Network of institutions engaged in sodium channel research.

Researchers from 102 countries contributed to the development of sodium channel research. Stratified by world region, north America had the greatest share (43.01%), followed by the European Union and Asia (Figure 4(a)). The United States published the most papers (1595, 39.77%), followed by China (578, 14.41%) and Germany (352, 8.78%). The geographic distribution of publishing countries is presented in Figure 4(b). The data showing the publication contributions of different countries and institutions is shown in Table 3, while the connection between countries is shown in the network (Figure 5(a)).

**Table 3.** The top 10 countries and institutions contributed to publications on sodium channel research.

Rank	Country/Territory	Frequency (%) N = 3684	Institution	Frequency (%) N = 3684
1	USA	1595 (43.30)	Yale University	73 (1.98)
2	Peoples R China	578 (15.69)	Emory University	68 (1.85)
3	Germany	352 (9.55)	UCL	61 (1.66)
4	England	348 (9.45)	Harvard University	59 (1.60)
5	Japan	259 (7.03)	Vanderbil University	57 (1.55)
6	Canada	201 (5.46)	University Calif San Francisco	56 (1.52)
7	Australia	182 (4.94)	Johns Hopkins University	54 (1.47)
8	France	182 (4.94)	University Queensland	52 (1.41)
9	Italy	171 (4.64)	University Pittsburgh	50 (1.36)
10	Netherlands	143 (3.88)	University Utah	46 (1.25)

Figure 5(b) shows that most of the publications were published by American institutions (Figure 5(b)), with Yale University producing the highest number of publications on sodium channels (73), followed by Emory University (68) and University College London (61) (Table 3).

### Active journals

In total, 313 journals have published more than four papers in the sodium channel field, comprising approximately 23.89% of all the published literature in this field. The top 10 journals in terms of number of publications are indicated in Table 4. The journal Plos One had the highest number at 170 (4.615%) (IF = 2.806, 2016), while Scientific Reports published 85 papers (2.307%) (IF = 4.259, 2016) on sodium channels. The Journal of Biological Chemistry ranked third at 73 papers (1.982%) (IF = 4.125, 2016).

**Table 4.** The Top 10 journals that published articles on sodium channel research.

Rank	Journal	Frequency (%) N = 3684	IF 2016	Country Affiliation
1	Plos One	170 (4.62)	2.806	United State
2	Scientific Reports	85 (2.31)	4.259	United State
3	Journal of Biological Chemistry	73 (1.98)	4.125	United State
4	American Journal of Physiology Renal Physiology	63 (1.71)	3.611	United State
5	Proceedings of The National Academy of Sciences of The United States of America	62 (1.68)	9.661	United State
6	Journal of Neuroscience	40 (1.09)	5.988	United State
7	Channels	38 (1.03)	2.042	United State
8	Epilepsia	38 (1.03)	5.295	United State
9	Journal of general Physiology	36 (0.98)	4.200	United State
10	Parasites Vectors	33 (0.90)	3.035	England

**Figure 6.**
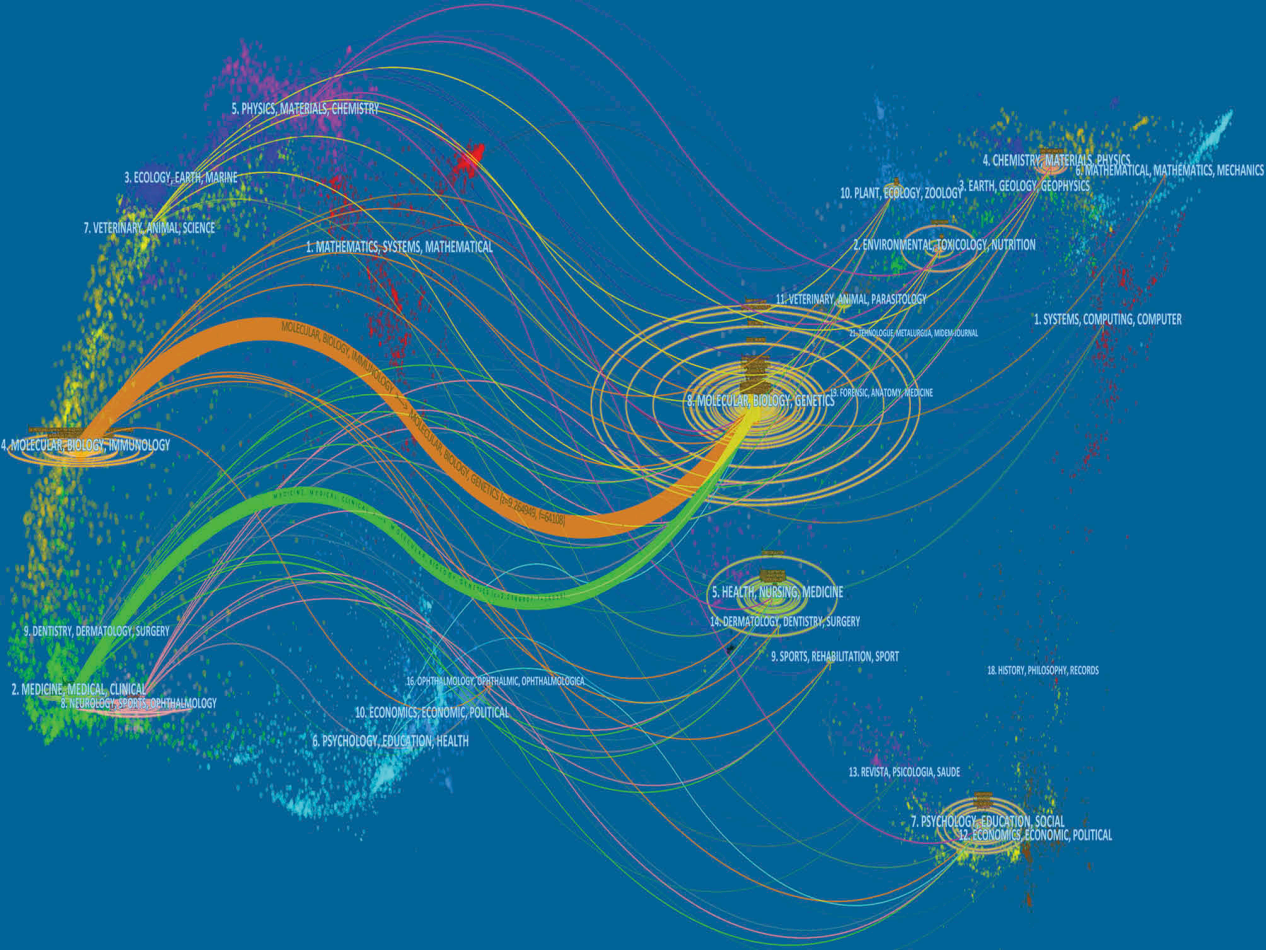
The dual-map overlay of journals related to sodium channel research.

A dual-map overlay of journals is presented in Figure 6. This map overlay indicates that most papers were published in mathematics journals, medical journals, medicine journals, clinical journals, molecular journals, immunology journals, and biology journals. They mainly cited journals in the areas of molecular research, biology, genetics, psychology, toxicology, and nutrition.

### Active authors, co-cited authors, co-cited references, and co-cited references on sodium channels

**Figure 7.**
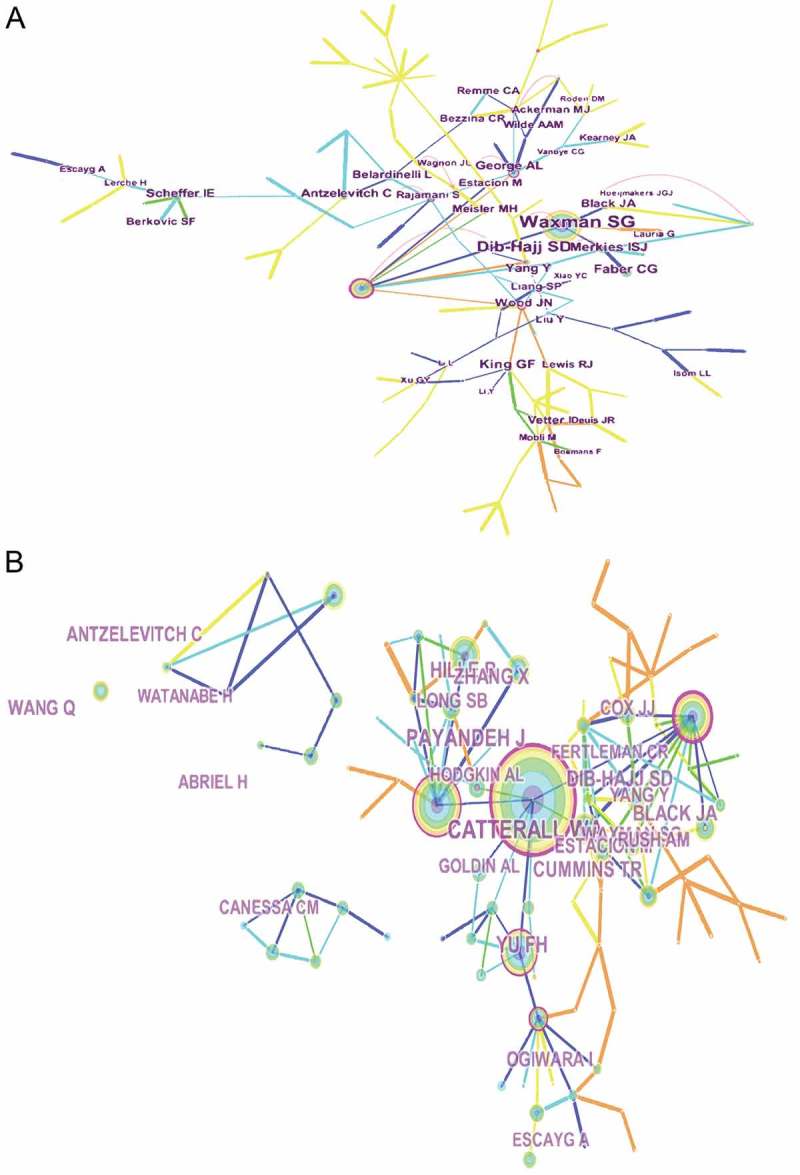
The analysis of authors. (a) Network of authors contributed to sodium channel research; (b) Network of co-cited authors engaged in sodium channel research.

Approximately 22,760 authors contributed over 4175 articles related to sodium channel biology. The networks shown in Figure 7(a) indicate the cooperation among authors. S.G. Waxman was the most prolific in terms of publications on sodium channels (62 papers), followed by S.D. Dib-hajj (34 papers) and K. Dong (26 papers).

There was also a wide distribution of co-authors in the field of sodium channels. The connection network between co-authors was measured using CiteSpace V (Figure 7(b)). The papers published by W.A. Catterall had the highest number of co-citations (585 papers), followed by S.D. Dib-hajj (325 papers) and J. Payandeh (319 papers).

Payandeh J., 2011, Nature, v475, p353 ranked first in terms of co-cited article references at approximately 205. This was followed by Catterall W.A., 2005, Pharmacol Rev, v57, p397 which was co-cited 116 times, followed by Catterall W.A., 2000, Neuron, v26, p13 (108) (see Table 5).

**Table 5.** The Top 10 authors, co-cited authors, and co-cited references on sodium channel research.

Rank	Author	Frequency (%) N = 3684	Co-cited Author	Frequency (%) N = 3684	Co-cited Reference	Frequency (%) N = 3684
1	Waxman, S.G.	62 (1.68)	Catterall, W.A.	585 (15.88)	Payandeh J., 2011, Nature, v475, p353	205 (5.56)
2	Dib-hajj, S.D.	34 (0.92)	Dib-hajj, S.D.	325 (8.82)	Catterall W.A., 2005, Pharmacol Rev, v57, p397	116 (3.15)
3	Dong, K.	26 (0.71)	Payandeh, J.	319 (8.66)	Catterall W.A., 2000, Neuron, v26, p13	108 (2.93)
4	Yang, Y.	25 (0.68)	Hille, B.	217 (5.89)	Zhang X., 2012, Nature, v486, p130	102 (2.77)
5	Eaton, D.C.	23 (0.62)	Antzelevitch, C.	208 (5.65)	Payandeh J., 2012, Nature, v486, p135	102 (2.77)
6	Liu, Y.	23 (0.62)	Black, J.A.	204 (5.54)	Cox J.J., 2006, Nature, v444, p894	95 (2.58)
7	Zhorov, B.S.	22 (0.60)	Cummins, T.R.	188 (5.10)	Yu F.H., 2006, Nat Neurosci, v9, p1142	78 (2.12)
8	Catterall, W.A.	21 (0.57)	Yu, F.H.	163 (4.42)	Ogiwara I., 2007, J Neurosci, v27, p5903	73 (1.98)
9	Du, Y.Z.	20 (0.54)	Long, S.B.	156 (4.23)	Canessa C.M., 1994, Nature, v367, p463	69 (1.87)
10	Tytgat, J.	20 (0.54)	Waxman, S.G.	153 (4.15)	Catterall W.A, 2012, J Physiol-London, v590, p2577	68 (1.85)

### References in publications on sodium channels

**Figure 8.**
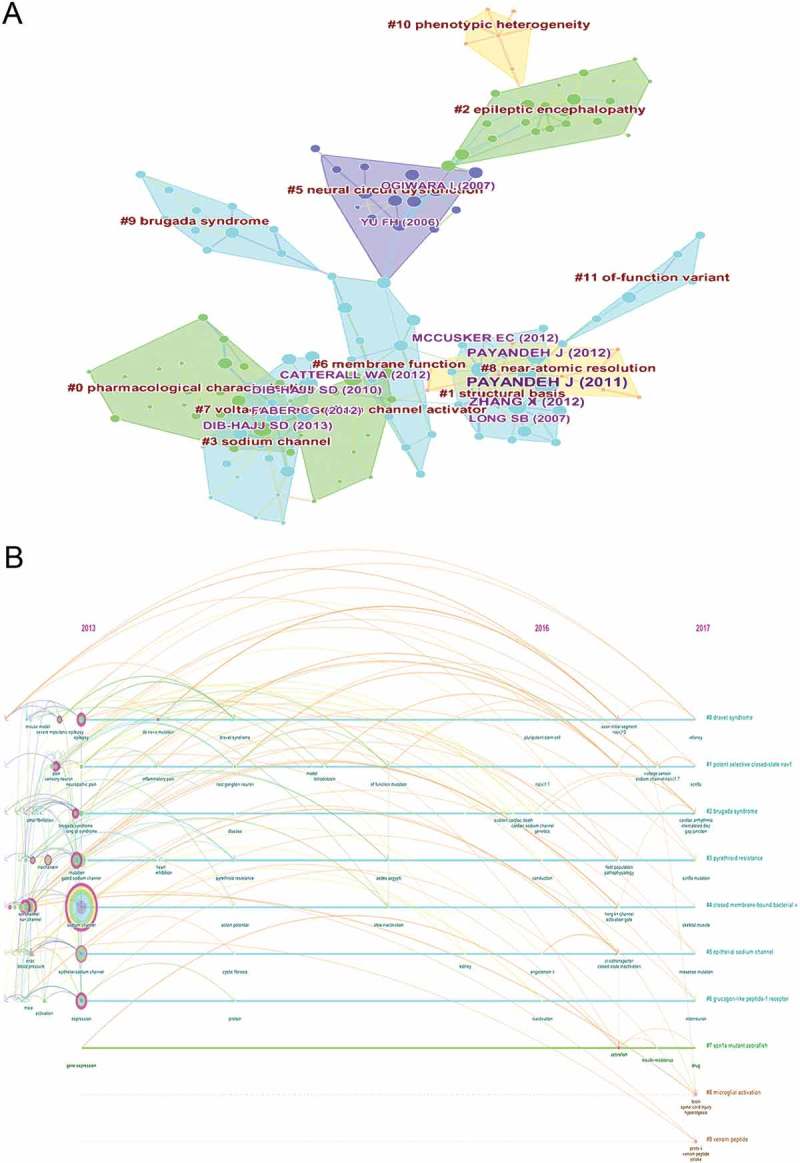
The analysis of references. (a) Co-citation network of references from publications on sodium channel research; (b) Co-citation network (timeline) of references from publications on sodium channel research.

One of the most essential bibliometric indicators is reference analysis. The top 2000 articles were selected for analysis by CiteSpace V. The co-citation network of references presented the publications’ scientific relevance (Figure 8(a)). The modularity Q score was equal to 0.8262 (>0.5) (Supplementary Figure 5), which meant that the network was reasonably divided into loosely coupled clusters. Moreover, the mean silhouette score exceeded 0.5 (0.5281) (Supplementary Figure 5), demonstrating that the homogeneity of the cluster was acceptable on average [16,17,18]. Clusters were labeled with index terms obtained from the references. The most massive cluster #0 was labeled “Dravet syndrome,” the second largest cluster #1 was labeled “Potent selective closed-state Na_V_1,” followed by cluster #2, the third largest, which was labeled “Brugada syndrome.” All mentioned clusters are also indicated in the timeline view (Figure 8(b)).

### Frequently encountered terms

**Figure 9.**
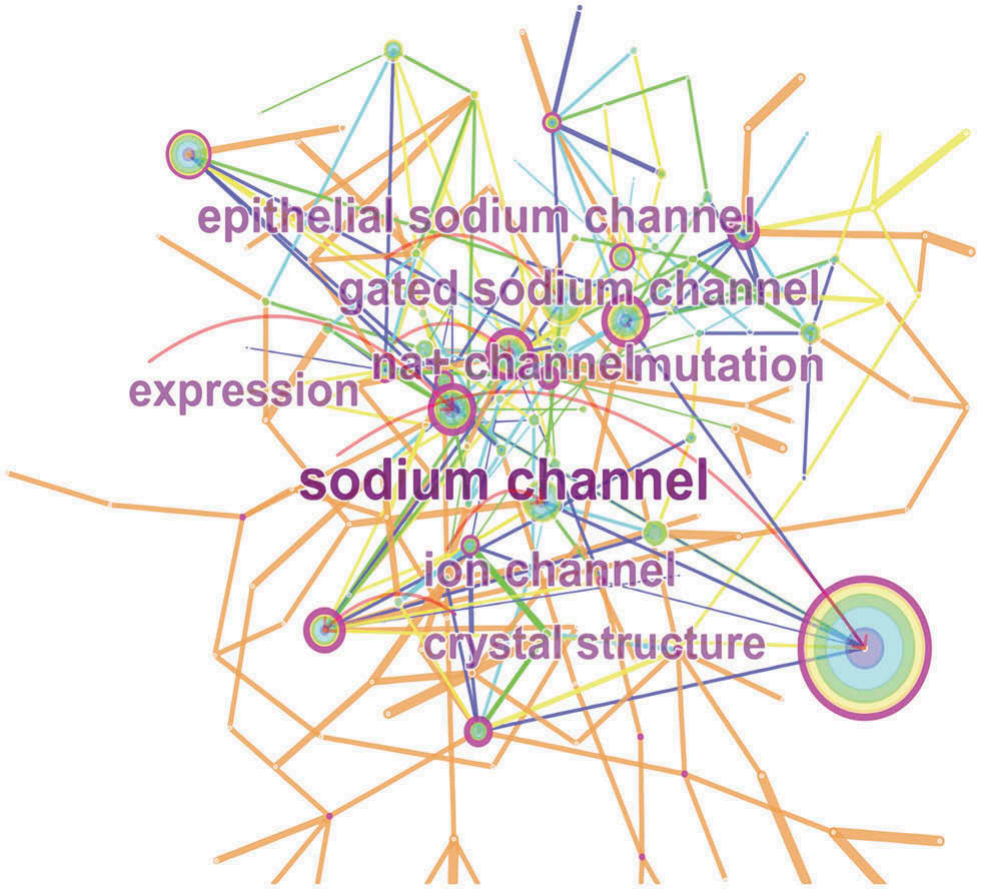
The analysis of keywords on sodium channel research.

**Figure 10.**
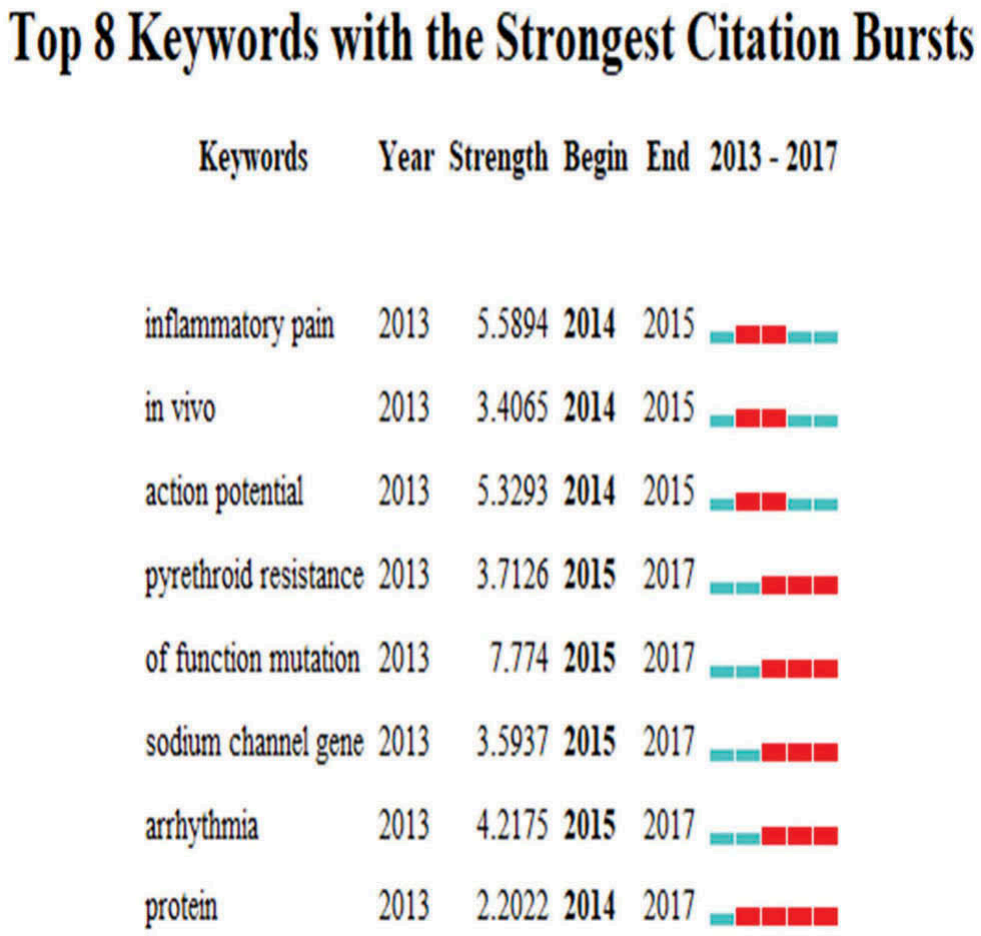
The keywords with the strongest citation bursts of publications on sodium channel research.

**Figure 11.**
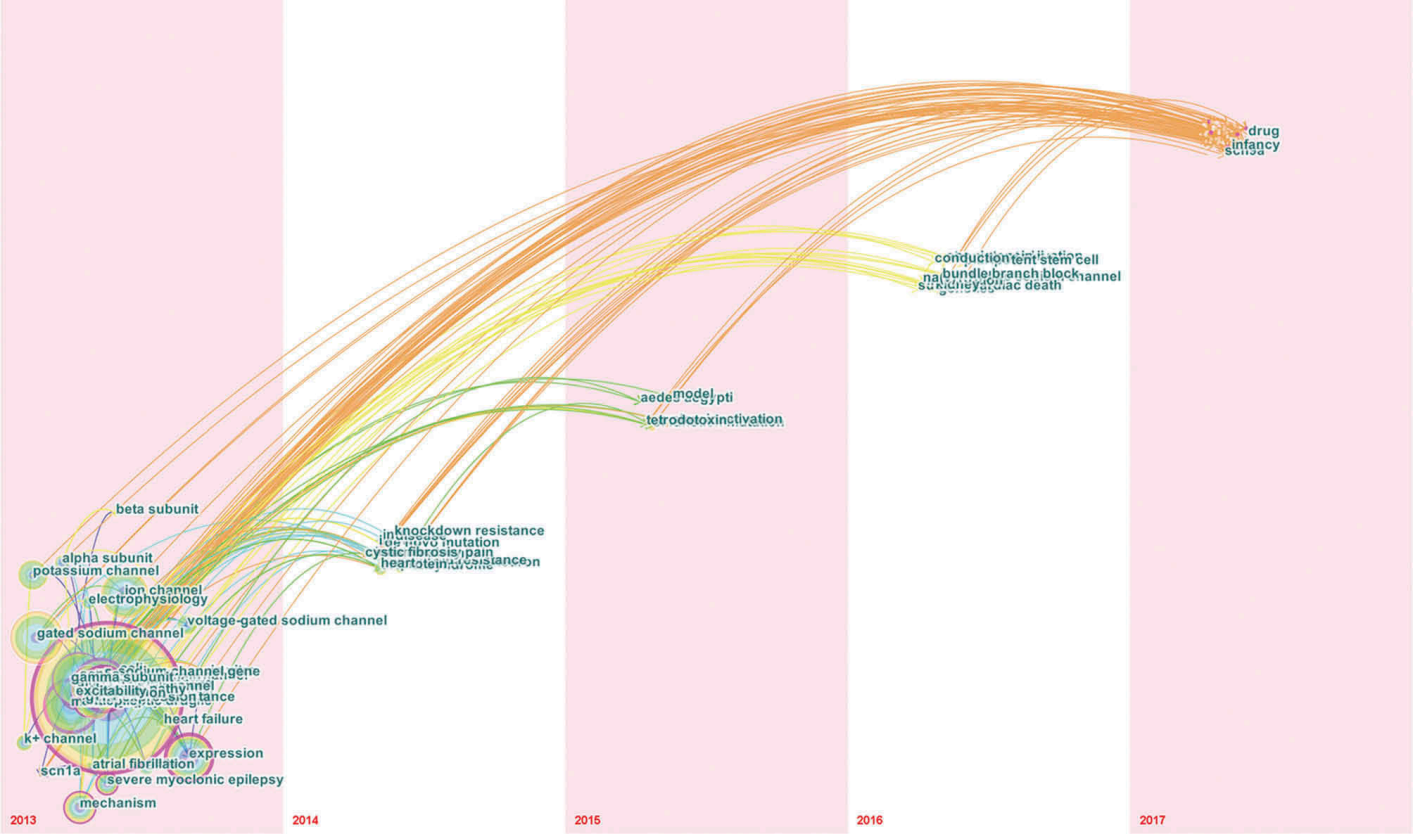
Keywords network (time zone) from publications on sodium channel research.

By extracting the 3684 relevant publications using CiteSpace V (Figure 9), keywords were identified and analyzed using strong citation bursts (Figure 10). The keywords that had strong bursts after 2014 were as follows: “pyrethroid resistance” (2015–2017), “of function mutation” (2015–2017), “sodium channel gene” (2015–2017), and “arrhythmia” (2015–2017). The time zone of keywords’ appearance and connections is also presented in Figure 11.

## Discussion

Our data regarding trends in publication year were consistent with two phases. The first phase (2013–2014) can be considered a popular period for sodium channel research, while the second phase (2015–2017) can be considered a stable period. Undoubtedly, the USA made significant contributions to sodium channel research, while China gradually came to pay more attention to this field.

However, journal impact factor had several limitations in terms of sodium channel development; for example, only the Proceedings of The National Academy of Sciences of The United States of America (IF2016, 9.661) had the highest impact factor of over 6.000; the Journal of General Physiology (IF2016 5.988) and Epilepsia (IF2016 5.295) had an impact factor of between 5.000 and 6.000; finally, just five journals, comprising Scientific Reports (IF2016 4.259), Journal of General Physiology (IF2016 4.200), Journal of Biological Chemistry (IF2016 4.125), American Journal of Physiology-Renal Physiology (IF2016 3.611), and Parasites Vectors (IF2016 3.035), had an impact factor of greater than 3.000. Moreover, journals with high impact factors exceeding 3.000 published just 11.672% of all papers (IF > 8.000, 1.683%; 8.000 > IF > 5.000, 2.117; 5.000 > IF > 3.000, 7.872). Overall, therefore, it is a challenge to publish more high impact factor papers on sodium channel research.

We found that the United States maintained the dominant position in terms of publications, citation frequency, and H-index in respect of which it has undoubtedly demonstrated both a high quantity and quality of sodium channel research papers. In contrast, Chinese publications had an advantage in terms of publication numbers (14.41%, 578), and with its citation and H-index at fourth and fifth respectively, it was the only developing country in the top 10 (5 European countries, 2 American countries, 3 Asian-Pacific countries). A strong collaboration among countries can also stimulate research and strong collaborations were found between the United States, China, Australia, Japan, and South Korea; between France and Spain; and between England and Germany. There collaborations increased published paper and this also improved studies on sodium channels.

Otherwise, except for University College London in England and the University of Queensland in Australia, the other eight institutions in the list of the top 10 were all from the United States. US institutions made up the largest proportion in the collaboration network, perhaps due to the fact that the United States had the highest number of publications.

Similarly, a previous bibliometric research study indicated that the left part of the dual-map overlay of journals represented the citing-journal, whereas the right part represented the cited-journal. The label presented the disciplines of the above journals. The citation-links lines, connecting the cited-journal and citing-journal and pointing to the right map, demonstrate their relationship.

Of the top 10 authors identified in this analysis, each contributed to more than 20 papers. Consequently, they were verified as “prolific authors.” However, only three of these prolific authors were included in the top 10 co-cited authors. Annual co-citation is an essential factor in presenting the quality of authors’ work, suggesting that prolific authors should focus on the quality rather than the quantity of their publications [16,17]. The number of citations of the top 10 co-cited authors was at least 153, and included the following: W.A. Catterall, who provided an overview of research using a combination of physiological, biochemical, molecular biological, and structural biological approaches, which expounded the function and structure of sodium channels at the atomic level [4]; S.D. Dib-hajj, who reviewed the contribution to pain of sodium channel isoforms (Na_V_1.1, Na_V_1.2, Na_V_1.3, Na_V_1.5, Na_V_1.6, Na_V_1.7, Na_V_1.8, and Na_V_1.9) [19]; and J. Payandeh, who reported the wild-type Na_V_Ab channels’ crystallographic snapshot from *Arcobacter butzleri*, which was captured in two potentially inactivate states [20].

The top 10 co-cited references numbered at least 68. They included Payandeh, J., 2011 Nature, v475, p353, which was claimed to be the representative paper of this author and ranked the first amongst co-cited references. This was followed by Catterall W.A., 2005, Pharmacol Rev, v57, p397, which introduced an overview of allostery as applied to receptor families and approaches for detecting, as well as providing recommendations for, the nomenclature of allosteric ligands and their properties and validating allosteric interactions [21]. The third-ranked author was Catterall W.A., 2000, Neuron, v26, p13, who reviewed the early sodium channels’ biochemical studies, and focused mainly on the molecular mechanisms of sodium channel regulation and function, as well as providing a prospective for future research on sodium channel proteins [21]. Our findings suggest that S.G. Waxman, S.D. Dib-hajj (Yale University School of Medicine, the United States), K. Dong (Renmin University of China, China), Y. Yang (Shanghai Institution of Materia Medical, Chinese Academy of Science, China), and D.C. Eaton (Emory University School of Medicine, the United States) might be good candidates for research collaboration in this field.

The frontiers of sodium channel research were predicted using the strongest citation bursts of publications. CiteSpace V captured the keywords that were identified as research frontiers over time. The top four research frontiers of sodium channel research were as follows:

Pyrethroid resistance: Pyrethroid resistance, a kind of mosquito-borne disease [22], was claimed to be mainly caused by recurrent space spraying interventions, or Impregnated Nets (ITNs) and Indoor Residual Spraying (IRS) coverage [23–26]. When involving non-synonymous mutations of the gene encoding the paratype VGSC (Soderlund, 2008), VGSC expresses in the central nervous system of the insect majority targeted by pyrethroids[26] and many insecticides use this idea as the principle. These mutations are called knockdown resistance (Kdr) and can be selected and used to disorder insect VGSC by conferring cross-resistance to the Dichlorodiphenyltrichloroethane (DDT) [26,27].

Function mutation: Most of the functions that depended on voltage-gated sodium ion channels were significant to the generation of action potentials[28]. Function mutation can disorder the action potential by regulating the sodium ion channel. The research demonstrated that biallelic loss-of-function mutations in SCN9A encoded sodium ion channel Na_V_1.7, causing an inability to experience pain [29]. Meanwhile, SCN11A encoding Na_V_1.7 functions as a relay station of the pain signal electrical transmission [19,30].

Sodium channel gene: RNA interference (RNAi) had shown potential for selectively regulating sodium channels by changing gene function; this has been used in controlling agricultural insect pests [31].

Arrhythmia: Cardiac arrhythmia modified the cardiac paralogue of the voltage-gated sodium channel by mutations in SCN5A [32]. It was demonstrated that the SCN5A-DKPQ mutation as a Long QT syndrome (LQTS) gene increases the persistent (or late) Na^+^ inward current to produce the LQTS phenotype, thereby prolonging the action potential duration [33].

Data on sodium channel publications were collected and retrieved from the Web of Science Core Collection (WoSCC) database (Science Citation Index-Expanded journals). WoSCC is superior at providing detailed data (e.g. annual publications, author information, journal sources, country and institution information), which are not provided by Google Scholar or PubMed. Thus, the data analysis was relatively sophisticated and objective. Furthermore, more than 95% of publications on the WoSCC are written in English. However, one limitation of our bibliometric analysis was that, compared with papers published several years ago, some recent articles did not have a high citation count. Nevertheless, bibliometrics can prove a useful tool for future research into sodium channels.

## Conclusions

Our study has demonstrated that numerous countries have focused on sodium channel research and made obvious contributions. Bibliometric analysis of the literature on the sodium channels of nerve system drugs is important in allowing clinicians and researchers to predict the frontiers of research. Junior researchers may use such analyzes to ascertain the top-cited articles, co-authors, leading authors, and journals. Clinicians, meanwhile, may use such analyzes to discover more about the mechanisms of nerve system disease drugs. Mapping the network of countries, institutions, and keywords can also be used to identify potential research cooperations. Despite the importance of the topic, the extent of annual publications should be focused on and encouraged. Organizations can refer to this article as a reference when deciding whether or not to provide repeat funding to a given research team. In the meantime, institutions should integrate their research fields on sodium channels.
