# Supplementary material for: Bibliometric analysis of recent sodium channel research
Source: Channels (Austin). 2018 Sep 29;12(1):311–25. doi: 10.1080/19336950.2018.1511513 (PMC6986798; doi:10.1080/19336950.2018.1511513)
Supplement: Supplemental Material [file kchl-13-01-1511513-s001.zip › Figure 5 caption.docx]

Figure 5. The analysis of countries and institutions. A. Network of countries/territories engaged in sodium channel research; B. Network of institutions engaged in sodium channel research.

Figure 5. The analysis of countries and institutions. A. Network of countries/territories engaged in sodium channel research; B. Network of institutions engaged in sodium channel research.
