# Supplementary figures and images for: Bibliometric analysis of recent sodium channel research
Source: Channels (Austin). 2018 Sep 29;12(1):311–25. doi: 10.1080/19336950.2018.1511513 (PMC6986798; doi:10.1080/19336950.2018.1511513)

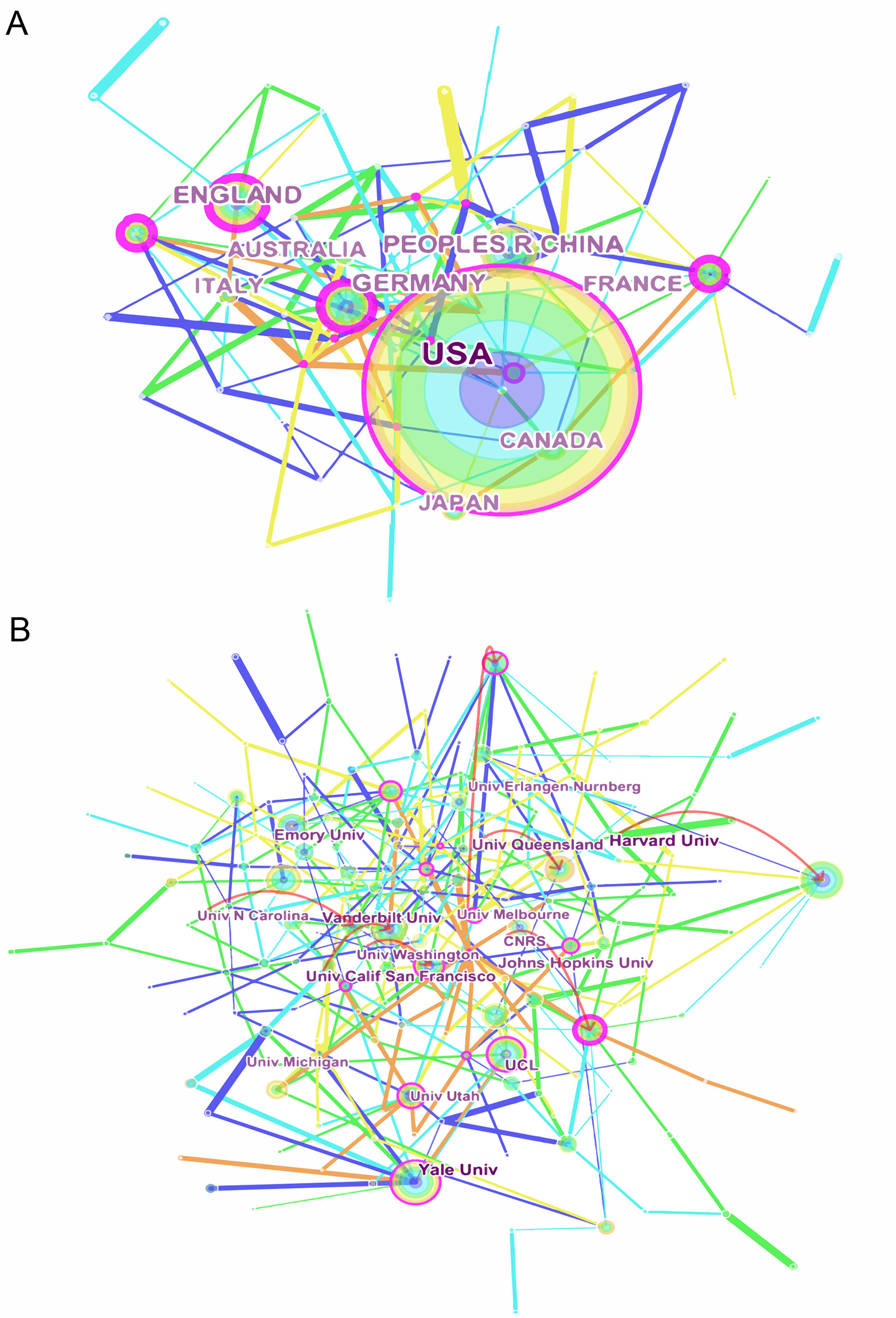

Supplement: Supplemental Material [file kchl-13-01-1511513-s001.zip › Figure 5 20180701.tif]
